# An Account of a Dropsy Cured by Blue Vitriol

**Published:** 1781-04

**Authors:** 


### SECTION II.

### ESSAYS AND OBSERVATIONS.

I. *An Account of a Dropſy cured by Blue Vitriol. By* William Wright, *M. D. Fellow of the Royal College of Phyſicians at Edinburgh, and of the Royal Society of London. Read* April 9, 1781.

STEPHEN Friar, a native of the iſland of Madeira, aged about twenty-four years, was ſteward of a ſhip from London to Jamaica. Soon after his arrival at Montego-Bay, he was taken ill of a fever, and left aſhore at ſick-quarters. Captain Mercer of Liverpool offered him a paſſage, and he was brought on board July 30, 1777, in a very low condition. The account he gave me was as follows:

That about the beginning of June he was ſeized with a fever, which, notwithſtanding the many medicines given him, did not entirely leave him till about ten days before he embarked. He complained of tightneſs about the præcordia, and of a difficulty of breathing when he walked. He had pains in his hips and limbs, was ſometimes much griped, and once in three or four days days had a few watery ftools, which fenfibly diminifhed his ftrength. His appetite was tolerable, his urine high coloured, and in fmall quantity. I ordered him fome ftomachic bitters, a nourifhing diet from the cabin, and to ftir about upon deck in fine weather.

Auguft 22d, he complained much of the pain in his ftomach, and of a difficulty of breathing when he attempted to walk upon deck. On a fuppofition that he might have vifceral obftructions, I gave him two grains of *merc. dulc.* combined with half a grain of *extract theb.* at bedtime two fucceffive nights, by which he was a little relieved, and returned to the ufe of the bitters.

Auguft 29th, a heavy gale of contrary winds came on, the veffel fhipped much water, and our patient being badly lodged, got wet in the night: this occafioned a fever with head-ach, thirft, &c. which however went off by the ufe of antimonial wine and laudanum; and he again took the bitters as before.

Sept. 1ft.—Although this man's appetite was for the moft part good, yet inftead of mending, he daily felt himfelf weaker; the tightnefs about the præcordia and difficulty of breathing increafed, and he appeared bloated in the face. I

now obferved his legs fwelled about the ancles, which retained the impreffion of my finger for a confiderable time. The fcrotum was clear and full of water, but no fluctuation could be felt in the abdomen.

I was at a lofs whether to afcribe this beginning dropfy to difeafed vifcera, or to a general debility of the fyftem. The former opinion prevailed, five grains of calomel, and three grains of extr. theb. were made into pills, which being divided into three dofes, one dofe was taken every night.

Sept. 5.—The fwelling in the legs and fcrotum rather increafed, and there was now an evident fluctation of water in the abdomen. On fearching the medicine box, I at firft found nothing that fuited my purpofe, either as a diuretic, or tonic.

During my refidence in Jamaica, I had often heard of the fuccefs of a noftrum in dropfy ufed by a furgeon in Montego-Bay. A friend procured me fome of the powder, and on examining it with the microfcope, and tafting it, I found it to be compofed chiefly of wild cinnamon and Roman vitriol; the latter feeming to be in no fmall quantity in a dofe. Neceffity now made me me determine to try this doubtful remedy rather than none at all.

℞ Vitrioli cerulei, Corticis Winterani occidentalis, utriusque ʒj. f. pulvis subtilissimus, cui adde mucilaginis G. Arabici q. s. ut fiat massa Pilularum, de quâ formentur pilulas N° xxiv. Capiat j. omni nocte horâ somni.

Sept. 6.—He had been griped a little in the night, and had two watery stools this morning, which probably would have happened, whether he had taken any medicine or not. I gave him half a grain of extr. theb. with the pill.

Sept. 7.—He passed more urine, and found himself rather easier. He continued to take a pill night and morning, and to repeat the opiate at bed-time.

Sept. 9.—During the last two days he passed abundance of urine, and had two loose stools a day. The size of the abdomen, scrotum, and legs greatly diminished. He walked upon deck with greater freedom, and his keen appetite was gratified with whatever the cabin afforded. Since he began the use of this medicine, he was directed to drink as often as he felt himself thirsty.

12.—The

12.—The weather being ſtormy he omitted his pill at bed-time, and had four watery ſtools in the night, which fatigued him a little. The ſwellings were entirely gone. He had ſome mutton broth for dinner, and ſeveral glaſſes of mulled Port-wine through the day; at bed-time the pill and opiate were repeated.

15.—The weather continuing bad; he had no medicine after the 12th, but the ſwellings had not returned; and as his appetite continued to be good, he diſcontinued the uſe of his medicines.

Oct. 9.—The ſhip arrived ſafely in Liverpool harbour, and on the 15th I ſaw the patient in good health, employed as a waiter in a tavern.

From the ſucceſs of the above medicine in this and other caſes I have heard of, I am of opinion it will ſucceed in all dropſies that are not owing to a fixed cauſe, ſuch as ſchirroſities of the liver, ſpleen, meſentery, &c. and which of courſe will require a different treatment.

